# A survey of orthopaedic journal editors determining the criteria of manuscript selection for publication

**DOI:** 10.1186/1749-799X-6-19

**Published:** 2011-04-28

**Authors:** Caroline B Hing, Deborah Higgs, Lee Hooper, Simon T Donell, Fujian Song

**Affiliations:** 1 Department of Trauma & Orthopaedics, St George's Hospital, Tooting, UK; 2 Royal National Orthopaedic Hospital, Stanmore, UK; 3 School of Medicine, Health Policy & Practice, University of East Anglia, Norwich, UK; 4 Institute of Orthopaedics, Norfolk & Norwich University Hospital, Norwich, UK

**Keywords:** editor, orthopaedic

## Abstract

**Background:**

To investigate the characteristics of editors and criteria used by orthopaedic journal editors in assessing submitted manuscripts.

**Methods:**

Between 2008 to 2009 all 70 editors of Medline listed orthopaedic journals were approached prospectively with a questionnaire to determine the criteria used in assessing manuscripts for publication.

**Results:**

There was a 42% response rate. There was 1 female editor and the rest were male with 57% greater than 60 years of age. 67% of the editors worked in university teaching hospitals and 90% of publications were in English.

The review process differed between journals with 59% using a review proforma, 52% reviewing an anonymised manuscript, 76% using a routine statistical review and 59% of journals used 2 reviewers routinely. In 89% of the editors surveyed, the editor was able to overrule the final decision of the reviewers.

Important design factors considered for manuscript acceptance were that the study conclusions were justified (80%), that the statistical analysis was appropriate (76%), that the findings could change practice (72%). The level of evidence (70%) and type of study (62%) were deemed less important. When asked what factors were important in the manuscript influencing acceptance, 73% cited an understandable manuscript, 53% cited a well written manuscript and 50% a thorough literature review as very important factors.

**Conclusions:**

The editorial and review process in orthopaedic journals uses different approaches. There may be a risk of language bias among editors of orthopaedic journals with under-representation of non-English publications in the orthopaedic literature.

## Background

Clinicians in orthopaedic practice are increasingly the subject of revalidation and are expected to be able to critically appraise new innovations with evidence based medicine the cornerstone of medical practice [1]. The clinician relies on published literature and conferences to expand his knowledge base but there is little known on the criteria used by journal editors in selecting manuscripts for publication [2,3]. All clinicians exhibit bias in some form with the ethical principal of 'equipoise' difficult to uphold during the review process [4,5].

There is little published in the orthopaedic literature on the subject of publication bias. Okike et al investigated the influence of non-scientific factors on the acceptance for publication in the Journal of Bone and Joint Surgery (American Volume) and found that manuscripts were more likely to be accepted if they were from the United States or Canada, reported a conflict of interest related to a non-profit entity or were authored by an individual with 10 or more prior publications in frequently cited orthopaedic journals [6]. Editors play a vital role in the peer review process but little evidence exists to support the quality of published research due to the use of editorial peer review [7-9].

We developed a questionnaire to establish which factors influence an editor's decision to accept a manuscript and the criteria used in each journal's review process.

## Methods

A Medline search was used to identify all English and non-English language orthopaedic journals that were listed on Index Medicus in 2008. Seventy journals were identified and approached between December 2008 and March 2009 with a questionnaire in English via an email link or by post if no email address was listed for the principal editor [see additional file 1]. The questionnaire was designed in English as only 10% of journals were not published in English (Czech, French, Spanish, Polish, German and Italian). We anticipated that most of these journal editors would either speak English as a second language or have access to a translator. Non-respondents were approached again 2 months later then 6 months later. The editors were informed that the survey was confidential and that their responses would be anonymised.

The questionnaire design was based on a validated questionnaire designed by Radford et al to determine the criteria used by dental journals in assessing manuscripts [2]. A set of additional questions were used to determine the demographics of the editors questioned and the journals they represented. The additional questions included questions on age, gender, qualifications, primary institution of work and country of residence. The questionnaire was designed to determine which factors in the study, the manuscript and which author characteristics influenced an editor's decision to accept or reject a manuscript. Questions were also asked regarding the review process and the journal characteristics. If an editor represented more than one journal he was sent a questionnaire specifically for each journal that he edited. Each journals' website was also examined to determine the demographics of editors and journals that had not responded. Journal impact factors for 2008 were also compiled from the Thomson Reuters Journal Citation report published in 2009.

Responses were collected on an online database http://www.surveymonkey.com (Portland, USA) and analysed using Excel spreadsheets (Microsoft, Seattle, USA) and SPSS version 11.5 (Chicago, Illinois). Statistical significance was set at p < 0.05. The impact factor and language of responders was compared to non-responders using a Students t test. A post hoc analysis of error level for completed surveys was also performed (survey sample random calculator, http://www.custominsight.com).

## Results

Thirty editors responded representing a 42% response rate (error level 11.4%, 90% confidence intervals). One American editor sent his response from France and one sent his response from a different state. One editor expressed an interest in feedback from the survey and one editor noted he did not like the questions. The mean impact factor for responding journal editors was 1.42 and for non-responders 1.53 which was not significantly different (p = 0.67).

Ninety percent of editors of orthopaedic journals were male, 1% female and 9% could not be determined. Eighty-three percent of editors spoke English as a first language. Fifty-seven percent of editors were over 60 years of age with the remainder being 41 - 60 years old. Sixty-seven percent worked in a University Hospital with only 3% working in a district hospital. Seven percent of editors had retired.

Analysis of the country of origin of publications showed 50% of journals were published in the US, 40% in the UK, 7% were from the East and 3% from Latin America. Analysis of publishers showed that 23% of orthopaedic journals are published by Elselvier, 18% were independent, 17% by Springer-Verlag, 14% by Lipincott, 6% by SLACK and 3% by Saunders, 3% SAGE and the remaining 16% by other smaller publishers. Thirty-seven percent of journals publish 12 issues per year and 37% publish 6 issues per year. The remainder varied from 4 to 10 issues per year.

Analysing the review process showed that 59% used a review proforma, 52% anonymised manuscripts before reviewing and 76% had a statistician routinely review all studies. In 89% of journals the editor could over-rule the reviewer's final decision with 78% of referees allowed to see each other's reports. In 59% of journals 2 referees routinely reviewed a manuscript with 35% of journals using 3 referees routinely. Of those journals that anonymised the manuscripts prior to peer review, 36% of editors could guess the authors or institutions in less than 10% of cases.

The editors' opinion regarding the most important factors in a submitted study likely to influence a decision to accept a manuscript are summarised in table 1. The most important factor likely to influence acceptance was that the study conclusions were justified (80%). An appropriate statistical analysis (76%), study findings that could change practice (72%) and the level of evidence of a study (70%) were also deemed important factors in the study design. The editors surveyed felt that the most important factors in the manuscript that influenced acceptance were that the manuscript was understandable (73%), well written (53%) and that the literature review was thorough (50%), table 2.

**Table 1:** Ranked design factors that influenced an editor's decision to accept a study for publication.

Study design factors	Percentage score (very unimportant/slightly unimportant/indifferent/slightly important/very important)
The study conclusions are justified	80% very important
The statistical analysis is appropriate	76% very important
The study findings could change practice	72% very important
The level of evidence	70% very important
The study design (e.g. randomised controlled trial)	62% very important
The study is a 'hot topic	53% slightly important
The study complies with the journal's aim	48% very important
The study has a large sample size	45% slightly important
The study reinforces my beliefs	41% very unimportant
The study findings are unexpected	39% slightly important
The study findings are statistically significant	38% very important

**Table 2:** Ranked manuscript factors that influenced an editor's decision to accet a study for publication.

Manuscript design factors	Percentage score (very unimportant/slightly unimportant/indifferent/slightly important/very important)
The manuscript is understandable	73% very important
The manuscript is well written	53% very important
The literature review is thorough	50% very important
The references include papers from my journal	40% indifferent
There is no financial conflict of interest	37% slightly important

When asked to rate what factors regarding the authors most influenced an editor to accept a manuscript for publication, 48% of editors regarded an author who correctly followed the instructions to authors as a slightly important factor. They were indifferent to how distinguished the senior author was (41%) or whether the author was from a high quality institution (45%) and rated knowledge of the authors or authors' work as very unimportant (45%).

## Discussion

The 'gold standard' editorial process defined by the Cochrane Collaboration aims to produce studies that are appropriate to the publication medium, important, useful, original, methodologically sound, ethical and accurate [10]. This is the first study to investigate journal editor demographics and the factors influencing orthopaedic journal editors during the manuscript review process. Evidence based medicine relies on the journal review process to provide an unbiased representation of high quality studies [11]. Deleterious effects on health policy and clinical decisions can result from publication and related biases. A recent Health Technology Assessment found that studies with significant or positive results were more likely to be published and published earlier than those with non-significant or negative results [12]. Exclusion of non-English language studies was also found to result in a high risk of bias in certain research areas such as complementary and alternative medicine [12].

The questionnaire used was based on a previously validated questionnaire designed by Radford et al [2]. Additional questions and a review of journal websites and instructions to authors were used to provide additional information on journal demographics. The response rate was 42% with no significant difference in impact factor between responders and non-responders' journal. Whilst the error level was 11.4% with 90% confidence limits, this is the first study of its kind and represents an indication of journal editors' preferences for manuscript acceptance as well as their characteristics which has not previously been documented in the literature. Poor response rates are a potential source of bias since the confidence with which the survey findings can be accepted are a function of the achieved sample size [13].

A small sample size resulting from poor response rates exists if the responders differ from the non-responders in terms of key characteristics [13]. A comparison of impact factor between responders and non-responders showed no significant difference with p = 0.67. However comparison of language of publication showed 96% of responders edited publications in English with only 76% of non-responders editing publications in English (p = 0.03). This may indicated that non-response was due to an inability to interpret the questionnaire which was in English. This is a weakness in the study design indicating that the results may be subject to language bias.

Analysis of the demographics of orthopaedic journal editors showed that 90% were male. Gender bias in terms of editors, reviewers and authors has been studied in the literature. Gilbert et al retrospectively reviewed 1851 research articles submitted to JAMA in 1991 and found no bias based on reviewers' or editors' gender on the decision to accept or reject a manuscript [14]. A further study conducted by Caelleigh et al of 50 female and 50 male reviewers showed no bias when assessing an empirical study with two versions, one attributing a lower income in women to gender issues and the other attributing it to social learning factors [12,15]. However, other studies have confirmed the presence of gender bias in the publication process [16,17]. A Scandinavian study used a sham paper with either male or female authors to assess gender bias in a 1637 randomly selected Swedish physicians. Female authors were ranked higher than male authors. Male assessors were found to reflect no gender bias but female assessors upgraded female authors more than male authors [18]. A study of grant proposals has also shown gender bias to the detriment of female applicants [19].

Eighty-three percent of editors spoke English as a first language with 50% of journals published in the US and 40% in the UK. Geographical bias has been shown to influence publication. A recent study of non-scientific factors influencing acceptance of manuscripts to the American Journal of Bone and Joint Surgery showed that manuscripts submitted from countries other than the US or Canada were significantly less likely to be accepted (odds ratio 0.51, 95% confidence interval, 0.28 to 0.92; p = 0.026) [7]. A Scandinavian study sent a methodologically flawed sham study in English and Scandinavian versions to 180 Scandinavian reviewers. The reviewers considered the English version significantly better than the Scandinavian version (p < 0.05) [20].

Fifty-seven percent of editors were over 60 years of age with the remainder being 41-60 years of age. Seven percent of editors had retired. Data were not collected on editorial experience. On searching Medline there is no published study in the literature investigating the effect of editor's age on the quality of peer review and this is an area that would benefit from further study. A study of the natural history of peer reviewers has found that the majority of peer reviewers deteriorated over time at a gradual rate but it is unclear whether this can be extrapolated to editors [21].

Analysis of publishers showed that 23% of orthopaedic journals are published by Elsevier, 18% were independently published and 14% published by Lipincott. The effect of publishers on the editorial process is unknown and deserves further study as only 18% of orthopaedic journals are published independently. A previous survey of editorial independence at medical journals owned by professional associations showed that 70% of editors reported having complete or near complete editorial freedom, although many received modest pressure from their owners over editorial content. Forty-eight percent of journal's board of directors surveyed could hire and 55% could fire the editor indicating that editors may not be entirely independent from the influence of publishers [22].

Publishers can influence the speed of publication of a manuscript according to the system of review. The introduction of online systems for peer review is seen as advantageous by authors and editors rather than reviewers [23]. Some publishers provide support to reviewers with online websites and surveys to improve knowledge and quality of the peer review process which may in turn improve the quality of publications [24].

Our study showed that 59% of journals used a review proforma, with 52% anonymising manuscripts and 76% using a statistician to routinely review all studies. Jefferson et al studied the effect of guidelines on peer review at the BMJ and Lancet and found there was no impact of guidelines on the quality of submissions but they did help editors manage submissions [25]. Gardner et al investigated the statistical assessment of manuscripts submitted to the BMJ using a checklist and found the statistical quality of the papers improved during the peer review process [26]. Seventy-six percent of surveyed orthopaedic editors ranked appropriate statistical analysis as very important when deciding to accept a paper yet only 76% had a statistician routinely review papers. Previous studies have shown that a routine statistical review can improve the quality of methodology of studies [27]. The use of a statistical checklist may therefore be beneficial in improving the quality of papers during the review process in journals without a routine statistical review.

Blinding of reviewers and editors has been extensively investigated in the literature with conflicting results. Some studies have shown blinding improves the quality of reviews and provides more consistent results [28-30]. More recent studies have shown that blinding of reviewers makes no difference to the quality of reviews although there is a significant increase in reviewers refusing to give their opinions [31-33]. Furthermore the process of blinding is not infallible with reviewers able to guess the identity of authors in small highly specialised areas of study and those authors who self cite [34]. Our study showed that 36% of editors could guess the author or institution in less than 10% of submitted anonymised manuscripts.

Our study found that in 59% of orthopaedic journals 2 referees were routinely used with 35% using 3 referees routinely. This is consistent with the review process of other biomedical journals with the majority using 2 reviewers and seeking the opinion of a third reviewer if the 2 reviewers contradicted each other or if a review was significantly delayed [23].

In 89% of orthopaedic journals surveyed the editor could over-rule the reviewer's final decision. Little is known about the editorial review process with previous surveys of editors of biomedical journals finding great diversity in editorial policy and procedures [35]. Recent studies of the editorial decision process have shown conflicting results. A study in the Netherlands found that individual editor's decisions were far from consistent with individual editor's decisions complying poorly to team decisions, editor's ratings not predicting reviewers' ratings and editor's ratings poorly predicting future citation [36]. A study from the US of editors at *Obstetrics and Gynaecology *was more encouraging with editors found to accurately assess the quality of manuscripts [37]. Early editorial screening of manuscripts without peer review can however decrease the time to publication and reduces the workload of peer reviewers without an effect on the quality of accepted manuscripts [38].

When orthopaedic journal editors were surveyed, the most important factors for acceptance of a manuscript were: justified study conclusions, appropriate statistical analysis, study findings that could change practice, an understandable and well written manuscript and that the instructions to authors were correctly followed. Studies of editors of biomedical journals have shown that manuscripts are more likely to be rejected if the topic lacks originality, is not suited to the journal's readership, has weak methodological quality and lacks impact to change current clinical practice [2,35]. Poor use of English is also cited as a reason for rejection [2]. Our study found no evidence of studies with positive results being more likely to be accepted for publication over those with negative or non-significant results which is in agreement with the current literature [39]. A study of editorial meetings at JAMA showed that clarity of a manuscript and response to referees' comments as well as journalism goals such as importance to medicine and strategic emphasis for the journal were also important in ensuring acceptance [40].

## Conclusions

This study represents the first to survey orthopaedic journal editors for their views on manuscript acceptance and the demographics of editors. Whilst the response rate was 42%, there was no significant difference in impact factor between responders and non-responders. Reasons cited by editors for manuscript acceptance are similar to those of other biomedical journals. Editor demographics indicate that most are male, over 40 years of age and speak English. There is an over-representation of UK and US based publications in the literature which increases the risk of language bias in the orthopaedic literature since poor English is cited as a reason to reject a manuscript and deserves further study.

## Competing interests

The authors declare that they have no competing interests.

## Authors' contributions

LH, FS and STD contributed to the study design. DH and CBH designed the questionnaire. CBH analysed the data and wrote the manuscript. All authors contributed to the manuscript.

## Supplementary Material

Additional file 1 **Survey Questionnaire**. The data provided represents the survey questionnaire Click here for file
