# Supplementary material for: A survey of orthopaedic journal editors determining the criteria of manuscript selection for publication
Source: J Orthop Surg Res. 2011 Apr 28;6:19. doi: 10.1186/1749-799X-6-19 (PMC3095562; doi:10.1186/1749-799X-6-19)
Supplement: Additional file 1 — Survey Questionnaire. The data provided represents the survey questionnaire [file 1749-799X-6-19-S1.PDF]

ortho journal editors

1.

We are conducting a survey to determine which factors influence your decision as an orthopaedic journal editor to accept or reject a manuscript.

This survey will take only 5 minutes of your time.

The School of Health, Policy & Practice
University Of East Anglia
Norwich
UK

1. Which factors in the STUDY influence your decision to accept a manuscript?

	very unimportant	slightly unimportant	indifferent	slightly important	very important
The level of evidence of a study					
The design of study (e.g. RCT)					
The study has a large sample size					
The statistical analysis is appropriate					
The study findings are statistically significant					
The study findings are statistically non-significant					
The study findings have important implications to changes in practice					
The study findings are unexpected					
The study is a ‘hot topic’					
The study reinforces my beliefs					
The study complies with the journal’s aim					
The study conclusions are justified					

2. Which factors in the MANUSCRIPT influence your decision to accept?

	very unimportant	slightly unimportant	indifferent	slightly important	very important
The manuscript is well written					
The manuscript is understandable					
The literature review is thorough					
The references include papers from my journal					
There is no financial conflict of interest					

3. Which factors about the AUTHORS influence your decision to accept a manuscript?

	very unimportant	slightly unimportant	indifferent	slightly important	very important
The author has correctly followed the instructions to authors					
I know the authors (or know the authors' work)					
The senior author is distinguished					
The author is from a high quality institution					

4. ABOUT THE REVIEW PROCESS:

	yes	no
Is there a review proforma?		
Is the manuscript anonymised?		
Does a statistician routinely review all studies?		
Can the editor over rule the reviewers' decision?		
Can referees see each other's reports before completing their own review?		

5. How many referees review a manuscript?

1 2 3 4

6. If anonymity is preserved, what percentage of papers are you able to guess the author or institution?

0% <10% 25% 50% 75% 100%

Optional comment

7. ABOUT YOU: what is your age?

20-40 41-60 >60

8. ABOUT YOU: what is your gender

male

female

9. ABOUT YOU: what are your qualifications?

10. ABOUT YOU: what is your primary institution of work?

- district hospital
- university hospital
- university
- private practice
- retired

Other institution

11. ABOUT YOU: what is your country of residence

12. ABOUT THE JOURNAL: how many issues are published per year?

1 2 3 4 5 6 7 8 9 10 11 12

13. ABOUT THE JOURNAL: what is the journal's most recent impact factor?

14. ABOUT THE JOURNAL: who is the publisher?

15. ABOUT THE JOURNAL: which country is it published in?

16. ABOUT THE JOURNAL: what is the language of publication?

- English
- French
- Spanish
- Italian
- German
- Polish
- Mandarin
- Cantonese
- Japanese

Other language

2. Thank you for completing the survey, your answers will be anonymised.
